# Trustworthy Tricksters: Violating a Negative Social Expectation Affects Source Memory and Person Perception When Fear of Exploitation Is High

**DOI:** 10.3389/fpsyg.2016.02037

**Published:** 2016-12-27

**Authors:** Philipp Süssenbach, Mario Gollwitzer, Laura Mieth, Axel Buchner, Raoul Bell

**Affiliations:** ^1^Department of Psychology, Philipps-University MarburgMarburg, Germany; ^2^Department of Psychology, Heinrich Heine University DüsseldorfDüsseldorf, Germany

**Keywords:** expectancy violation, fear of exploitation, memory, trustworthiness, victim sensitivity

## Abstract

People who are high in victim-sensitivity—a personality trait characterized by a strong fear of being exploited by others—are more likely to attend to social cues associated with untrustworthiness rather than to cues associated with trustworthiness compared with people who are low in victim-sensitivity. But how do these people react when an initial expectation regarding a target’s trustworthiness turns out to be false? Results from two studies show that victim-sensitive compared with victim-insensitive individuals show enhanced source memory and greater change in person perception for negatively labeled targets that violated rather than confirmed negative expectations (the “trustworthy trickster”). These findings are in line with recent theorizing on schema inconsistency and expectancy violation effects in social cognition and with research on the different facets of justice sensitivity in personality psychology.

## Introduction

Cooperation between individuals requires mutual trust. If person A is in dire straits and asks person B to lend him or her some money, then A should trust that B will not exploit A’s state of emergency, and B should trust that A will eventually pay the money back. Neither person can be sure that this is actually the case; this makes the described exchange fundamentally uncertain. This is the paradox of trust (Yamagishi, 2001): the more uncertain a situation, the more trust is required, but—at the same time—the more difficult it is to decide whether one’s interaction partner is actually trustworthy.

Humans have a fundamental aversion to being exploited by others. However, this aversion is stronger for some people than for others: People who are victim-sensitive harbor a latent fear of being exploited and react particularly strongly toward experiences of unfairness (Gollwitzer et al., 2005, 2013; Gollwitzer and Rothmund, 2009). Because the possibility of exploitation is aversive and present in many contexts, people are well advised to trust others only when there is reason to do so. Stated differently, whenever a specific social situation entails cues suggesting that one’s interaction partner is not trustworthy, then trust becomes riskier and, thus, less likely. It is therefore highly functional (in particular for people aversive to exploitation) to attend to cues that are informative about another person’s untrustworthiness, and research shows that people actually do use these cues before they make a trustworthiness decision (e.g., Zebrowitz and Montepare, 2008; Gollwitzer et al., 2009; Todorov et al., 2009). Such cues include current behavioral cues such as the things a person says, the way he or she looks, or their facial expression, as well as information about a person’s past behavior, reputation, or background. Negative social labels, in particular, can be used to quickly form an impression about an interaction partner, and to guide social behaviors. However, these social labels may fail to do justice to each individual. A person who is said to be a trickster (i.e., a negative social label) is possibly regarded as untrustworthy, but may turn out to actually be a very nice and helpful person.

Therefore, it can be considered functional to attend to negative social labels because they provide a quick orientation in a complex social environment, but it is also important to remember information that is inconsistent with these labels, and to integrate it in one’s judgment. The present study examines what happens when an untrustworthiness-related cue turns out to be invalid: would victim-sensitive participants with their strong fear of exploitation be able to remember that a trickster turns out to be trustworthy? Or would they show an inflexible memory bias for untrustworthy behavior?

In Study 1 we will show that, perhaps counterintuitively so, victim-sensitive compared with victim-insensitive individuals indeed have a memory advantage for the trustworthy (but not for the untrustworthy) trickster. In Study 2 we will show that victim sensitivity also has an asymmetric effect on people’s person perception: Victim-sensitive compared with victim-insensitive participants update their trustworthiness perceptions about the trustworthy trickster more strongly than about the untrustworthy trickster, whereas the updating of another type of expectancy-inconsistent target (e.g., the untrustworthy scientist) is not influenced by victim sensitivity. These findings are incompatible with the notion of a “cheater detection module” (Cosmides, 1989), but they can be well explained by modern schema inconsistency and expectancy violation theories, as will be described in the following.

## Victim Sensitivity and Expectancy Violation

Victim sensitivity is a self-directed concern for justice characterized by a fear of being exploited. It predicts less pro- and more anti-social behavior (Gollwitzer et al., 2005). Past research has demonstrated that victim sensitivity is a highly stable personality trait (Schmitt et al., 2005) and has documented its location in the personality space (for the relationships with jealousy, just-world beliefs, or Machiavellianism, see Schmitt et al., 2005; for the relationships with the Big Five personality traits, see Schmitt et al., 2010). According to the Sensitivity to Mean Intentions (SeMI) model (Gollwitzer and Rothmund, 2009), victim-sensitive individuals are specifically sensitive to contextual cues that are associated with meanness, recklessness, and untrustworthiness. In social dilemma situations—that are typically characterized by some degree of uncertainty concerning one’s partners’ intentions—victim-sensitive individuals expect to be exploited and thus tend to defect (Gollwitzer et al., 2009). Hence, some of the uncooperative and anti-social behaviors displayed by people high in victim sensitivity can be understood as a means to protect themselves from (assumed) victimization.

Whereas previous studies have primarily focused (1) on the cognitive schemas (i.e., untrustworthiness expectations) that victim-sensitive individuals apply in social situations and (2) on the behavioral consequences of victim sensitivity in these situations, the present study will be the first to investigate the effect of being confronted with schema-incongruent information on source memory and person perception. In other words, the following questions will be addressed by the present studies: Do expectancy violations have a source memory advantage for victim-sensitive individuals? Are victim-sensitive compared with victim-insensitive individuals more influenced by a violation of positive or negative expectations? And do they update their cognitive schemas accordingly?

## Cheater Detection and Expectancy Violation

According to evolutionary psychologists, the analysis of evolutionary pressures is essential for understanding how the human mind works. One such pressure is the maintenance of social exchange within larger groups of non-kin, as cooperation between unrelated individuals is prone to be exploited by cheaters. Thus, the ability to identify and remember people who cannot be trusted is considered particularly adaptive (Cosmides and Tooby, 1989, 2005); hence, many authors argue in favor of the existence of a specialized cognitive module devoted to the detection of, and memory for, cheaters. Indeed, there is some empirical evidence that supports the assumption of a specialized “cheater detection module” (Mealey et al., 1996; Oda, 1997).

Other findings, however, speak against the existence of a specialized cheater detection module and are in favor of more general mechanisms (Bell and Buchner, 2012): in the area of memory research, participants usually show enhanced memory for the violation of both positive *and* negative expectancies (Barclay, 2008; Bell et al., 2010, 2012, 2015; Suzuki and Suga, 2010; Mieth et al., 2016). Thus, human memory is indeed adaptive, but even more strongly than suggested. In line with an evolutionary account, memory for cheaters is quite good in contexts in which cooperation is the norm and cheating is unexpected. However, this pattern flips when the context changes participants’ expectations. For example, in cooperation games with very low cooperation rates trustworthy individuals are particularly well remembered (Barclay, 2008; Bell et al., 2010). These findings suggest that enhanced cheater memory is best explained in terms of an expectancy violation or schema inconsistency account.

The schema-plus-tag model (Graesser and Nakamura, 1982), for example, states that memory discrimination for schema-consistent information is poor because schema-consistent information is always produced at test, whether it was actually present at encoding or not. According to this model, memory discrimination for the untrustworthy behavior of a trickster would be poor because untrustworthiness is already part of the negative stereotype of a trickster, and is copied into the memory trace (guessed), regardless of whether it was actually present at encoding or not. Schema-*inconsistent* information (e.g., a trustworthy behavior of a trickster), in contrast, is stored in memory in the form of tags.

It is therefore an open question how memory accuracy for different social targets is affected by victim sensitivity. How do victim-sensitive individuals process information that violates or confirms their negative social expectations about a particular target? Based on what is known so far, two patterns of results are conceivable. On the one hand, if the perception of, and memory for, cheaters was driven by experienced negativity (as the “cheater detection” literature would suggest), then individuals who are victim-sensitive should be more influenced in their judgment and have enhanced memory for cheaters (as they experience stronger emotional reactions toward learning that someone is truly untrustworthy) than individuals who are less victim-sensitive.

On the other hand, if the perception of, and memory for, cheaters was driven rather by schema inconsistency than by negativity (as the current state of memory research suggests), then a different pattern should be expected. Untrustworthy behavior is already a part of the negative stereotypes associated with negative social labels. Therefore it does not change one’s attitude toward that target and does not have to be remembered separately. Learning that a dubious target has acted truly trustworthily, however, comes as a much bigger surprise to people who fear exploitation than to those who do not, as the former hold much more negative expectations toward such targets in the first place. Thus, if individuals’ memory is particularly good for schema-inconsistent behaviors, then a stronger fear of exploitation should cause better source memory for targets *violating* negative expectancies than for targets *confirming* negative expectancies. Likewise, we would expect victim sensitivity to predict a greater change in the perception of targets violating negative expectancies than in the perception of targets confirming negative expectancies. Put more bluntly, victim-sensitive compared with victim-insensitive individuals should remember the untrustworthy behavior of a trickster particularly poorly because the untrustworthiness of this target is already part of the negative stereotype while the atypical trustworthy information about the trickster should be remembered particularly well. The current state of research on memory for cheaters and non-cheaters suggests that enhanced memory is most likely to be driven by schema inconsistency (see Bell and Buchner, 2012); thus, we consider the latter pattern of results to be more likely.

## Research Overview

The present research aims to investigate how victim-sensitive individuals compared with victim-insensitive individuals perceive and memorize targets with negative or positive social labels when these targets supposedly did something that was inconsistent with their respective label. Based on the theorizing presented above, it is predicted that victim-sensitive individuals hold more negative expectations toward targets associated with negative social labels, which should influence the classification of these targets in a subsequent memory test in two ways. First, victim-sensitive compared with victim-insensitive individuals should rely heavily on their biased expectations when memory is not available. In consequence, they should show a more pronounced bias toward guessing that targets with negative social labels were previously associated with untrustworthy behavior. Second, regarding memory accuracy, it is predicted that victim sensitivity is associated with enhanced memory for *violations*, but not for *confirmations*, of negative expectations. Third, it is predicted that victim-sensitive individuals are more likely to update their trustworthiness perceptions for negative expectancy violations, but not for negative expectancy confirmations.

Predictions regarding violations of *positive* expectations are less straightforward. Past research has shown that both victim-sensitive and victim-insensitive individuals react similarly to cues of trustworthiness (Gollwitzer et al., 2012). Hence, their expectations regarding targets with positive social labels (i.e., “scientist”) are not expected to differ. However, violations of these positive expectations might pose a greater threat to victim-sensitive individuals, thereby affecting memory and person perception more strongly. Thus, whereas initial expectations toward these targets should not be influenced by victim sensitivity (and thus a pure expectancy violation account would not predict an effect of victim sensitivity on memory and perception for such cases), it is conceivable that the trustworthiness violation itself is stronger for people high in victim sensitivity (which would imply an effect of victim sensitivity also in cases of a violation of positive expectations).

These hypotheses were tested in two studies. Study 1 examined the influence of victim sensitivity on source memory. To that end, participants viewed faces that were accompanied by a positive (e.g., scientist, firefighter) or negative social label (e.g., trickster, former prisoner). After a short delay this information was complemented with a behavioral description that represented either prosocial (i.e., trustworthy) or antisocial (i.e., untrustworthy) behavior. After viewing these profiles, participants completed a surprise source memory test in which they viewed faces and indicated whether a face had been presented before, and, if so, whether it was paired with trustworthy or untrustworthy behavior. It is predicted that participants high in victim sensitivity compared to participants low in victim sensitivity have more negative social expectations toward targets with negative social labels, which is reflected in a bias toward guessing (i.e., in the absence of memory about the correct behavior) that faces with negative labels are associated with untrustworthy behavior. This finding would be consistent with prior research showing that victim-sensitive individuals rely particularly strongly on untrustworthiness cues (Gollwitzer et al., 2012). Importantly, these negative social expectations should result in particularly good source memory for the *violation* of *negative* expectations; that is, for negatively labeled targets who displayed trustworthy (compared to untrustworthy) behavior.

In Study 2, participants’ perceptions of the targets’ trustworthiness were examined before and after they learned about the trustworthy or untrustworthy behavior of the positively or negatively labeled targets. Importantly, an experimental manipulation was included that aimed at making fear of exploitation salient to demonstrate that differential effects of victim sensitivity are indeed causally attributable to differences in people’s victim sensitivity. Thus, it is predicted that victim-sensitive participants in the fear of exploitation condition harbor more negative initial expectations toward targets with negative social labels than victim-sensitive participants in the control condition or victim-insensitive participants. Given that higher victim sensitivity should be associated with greater schema violation regarding dubious targets who show trustworthy behavior (compared to dubious targets who show untrustworthy behavior), changes in perceived trustworthiness should be stronger for “trustworthy tricksters” than for “untrustworthy tricksters” (i.e., selective updating). Importantly, these effects should be more pronounced in the fear of exploitation condition than in the control condition.

## Study 1

### Method

#### Participants

Participants were recruited from an undergraduate student pool of a large German university. One data set was removed because it turned out later the participant had participated twice in the experiment. The remaining sample consisted of 104 students (68 women; *M*_Age_ = 24, *SD*_Age_ = 4).

#### Materials

Ninety pictures (512 × 768 pixels) of frontal male faces with a neutral facial expression were selected from the FERET database (Phillips et al., 1998). We only used faces that had received neutral ratings of facial trustworthiness in a norming study (*M* = 3.28 on a scale ranging from 1 [not at all trustworthy] to 6 [very trustworthy]; *SD* = 0.22).

In a separate norming study, 15 participants (*M*_Age_ = 24, *SD*_Age_ = 2) rated the trustworthiness of 194 social labels using a scale ranging from 1 (not at all trustworthy) to 6 (very trustworthy). Out of these, 45 positive labels with a mean trustworthiness of 4.43 (*SD* = 0.25) and 45 negative labels with a mean trustworthiness of 1.92 (*SD* = 0.45) were selected as stimulus material. Examples for positive labels are “scientist,” “professor,” “firefighter,” and “ambulance driver;” examples for negative labels were “trickster,” “Satanist,” “former prisoner,” or “gang member.”

In yet another norming study, (*N* = 40, *M*_Age_ = 28, *SD*_Age_ = 10), behavioral descriptions of trustworthy and untrustworthy behaviors were rated on a scale ranging from -3 (very untrustworthy) to +3 (very trustworthy). The 25 descriptions of untrustworthy behavior had a mean rating of -2.22 (*SD* = 0.42), and the 25 descriptions of trustworthy behavior had a mean rating of +1.89 (*SD* = 0.45). An example for untrustworthy behavior is “He exploits the trust of older people and steals valuable items from their apartments.” An example of trustworthy behavior is “On his way home he once risked his life to rescue a kid that fell into a frozen pond.”

#### Procedure

In the encoding phase, participants saw 50 male faces. The faces were randomly assigned to 25 negative and 25 positive labels. Each trial started with the presentation of a face. Below the facial photograph, a label was presented (e.g., “F. D. is a scientist,” or “S. D. is a trickster”). After 4.5 s, face and label were complemented by a behavioral description. The behavioral descriptions were randomly selected with the restriction that the negative labels were paired with 15 untrustworthy and 10 trustworthy descriptions and the positive labels were paired with 15 trustworthy and 10 untrustworthy descriptions. Participants were required to rate the likeability of the person, and then initiated the next trial by clicking on a “continue” button. Negative and positive social labels were paired with more valence-congruent in comparison to valence-incongruent behaviors because people’s negative or positive stereotypes should not be blatantly disconfirmed by a high proportion of schema-inconsistent pairings in the encoding phase.

Immediately after the encoding phase, participants received instructions for a surprise source memory test, in which 80 facial photographs were presented in a random order. Each face was accompanied by a social label (i.e., “scientist,” “trickster”). Half of the faces had been presented in the encoding phase. Ten faces with negative labels had been described as untrustworthy, 10 faces with negative labels had been described as trustworthy, 10 faces with positive labels had been described as untrustworthy, and 10 faces with positive labels had been described as trustworthy. Of the 40 new faces, 20 were accompanied by negative labels, and 20 were accompanied by positive labels. The faces and labels were randomly selected to be presented in either the encoding phase or test phase. Faces and labels were randomly assigned to conditions.

Each test trial started with the presentation of a face with a label. After a 1.5 s interval, the likeability rating scale appeared (ranging from 1 [not at all likeable] to 6 [very likeable]). After rating the person’s likeability, the participants were asked to indicate whether the face was old or new (had been presented during the encoding phase or not). When the person had been classified as old, participants were asked to indicate whether the person was accompanied by a trustworthy or an untrustworthy behavior description during the encoding phase. After the test phase, participants completed a paper-and-pencil version of Schmitt et al.’s justice sensitivity questionnaire (Schmitt et al., 2010). Victim sensitivity was assessed with 10 items (Cronbach’s α = 0.82). Example items are “It bothers me when others receive something that ought to be mine” or “It makes me angry when others receive a reward that I have earned.” Each item was rated on a 6-point response scale ranging from 1 (not at all true) to 6 (absolutely true).

#### Design

A multinomial model was used to distinguish between old-new recognition, source memory, and source guessing. Given an estimated small effect of ϖ = 0.04 (estimated on the basis of pilot studies), α = 0.05, and 80 answers in the source memory test, an *N* of 104 is sufficient to detect an effect with a power of 1 – β = 0.95. Power was calculated using G^∗^Power (Faul et al., 2007).

### Results and Discussion

#### Source Memory and Source Guessing

**FIGURE 1:**
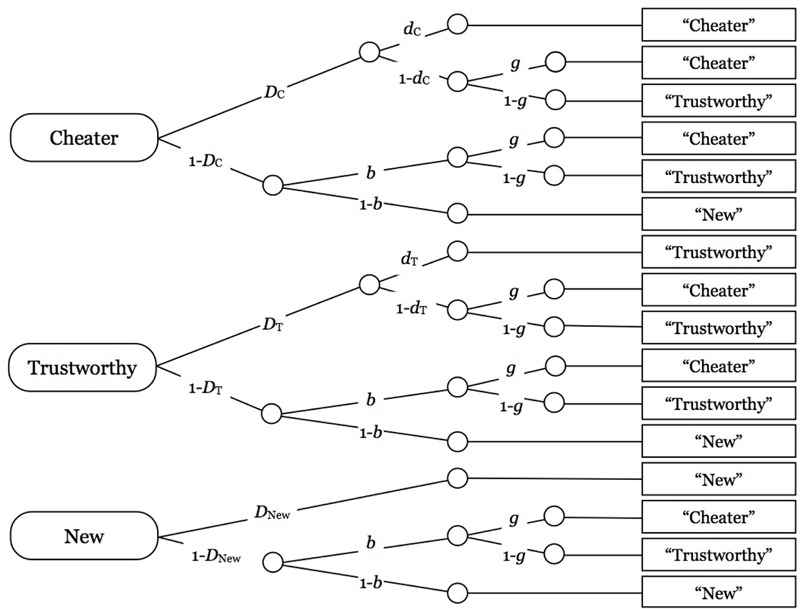
**Bayen et al.’s (1996) source memory model, adapted for the present study.** Rounded rectangles on the left side represent the stimulus persons (cheaters, trustworthy persons, and new persons). Letters along the branches represent the model parameters. *D*: probability that a person (a face with a label) is correctly recognized as old or new. *d*: conditional probability that the context (untrustworthy or trustworthy behavior) is correctly remembered. *b*: conditional probability of guessing that a person has been presented during the encoding phase. *g*: conditional probability of guessing that a person has previously been associated with untrustworthy behavior.

A multinomial source monitoring model (Bayen et al., 1996; see **Figure 1**) was used to distinguish between guessing, source memory, and old–new recognition. This model is well validated (Bayen et al., 1996; Erdfelder et al., 2009), and has been used in previous studies to disentangle the effects of schema (in-) consistency on source guessing and source memory (Bayen and Kuhlmann, 2011; Bell et al., 2015). The first tree represents the processing tree of a face-label pair that was paired with a description of untrustworthy behavior during the encoding phase. With probability *D*_C_, participants recognize the face-label combination as old. With the conditional probability *d*_C_, source memory, that is, memory for the association of the face-label combination with an untrustworthy behavior description, is correctly remembered, in which case the participant is able to correctly classify the person as a cheater. With the complementary probability 1 – *d*_C_, the participant has no source memory. In this case, the participant has to guess, with probability *g*, that the person was described as a cheater, or, with probability 1 – *g*, that the person was associated with trustworthy behavior. With probability 1 – *D*_C_, the participant fails to recognize the face-label combination as old, in which case participants guess, with probability *b*, that the item is old, or, with probability 1 – *b*, that the item is new. If the item is guessed to be old, participants further guess, with probability *g*, that the person was described as a cheater, or, with probability 1 – *g*, that the person was described as trustworthy.

The other two trees represent the processing of face-label combinations that were associated with trustworthy behavior descriptions and face-label combinations that were new (only presented at test), respectively. Model 5d of Bayen et al.’s (1996) taxonomy of identifiable submodels, which includes the restriction *D*_C_ = *D*_T_ = *D*_New_, was used^1^. Two sets of the processing trees displayed in **Figure 1** are needed for the analysis of the present data set, one for faces with negative labels, and one for faces with positive labels. Parameter estimations and goodness-of-fit tests were performed using multiTree (Moshagen, 2010).

The base model provided a good fit to the data, *G*^2^(2) = 0.05, *p* = 0.97. When no source memory was available, participants had a higher probability of guessing that a person had been described as a cheater when the social label was negative than when the label was positive, Δ*G*^2^(1) = 6.72, *p* < 0.01. The estimates of the source guessing parameter *g* and of the source memory parameter *d* are reported in **Tables 1** and **2**, respectively.

**Table 1:** Parameter estimates of the guessing bias parameter *g* representing the conditional probability of guessing that the person was a cheater rather than a trustworthy person as a function of label (Study 1).

Label	Parameter estimate	(*SE*)	[0.95 Confidence Interval]
Negative	0.61	(0.05)	[0.51 -0.71]
Positive	0.43	(0.04)	[0.35 -0.52]

**Table 2:** Parameter estimates of the source memory parameter *d* as a function of label and behavior (Study 1).

Label	Behavior	Parameter Estimate	(*SE*)	[0.95 Confidence Interval]
Negative	Untrustworthy	0.27	(0.11)	[0.05 -0.49]
Negative	Trustworthy	0.57	(0.05)	[0.48 -0.67]
Positive	Untrustworthy	0.38	(0.06)	[0.25 -0.50]
Positive	Trustworthy	0.48	(0.07)	[0.34 -0.62]

Consistent with previous studies (Mieth et al., 2016), participants showed an asymmetric expectancy violation effect. For faces with negative labels, source memory was better for the trustworthy behaviors in comparison to untrustworthy behaviors, Δ*G*^2^(1) = 4.74, *p* = 0.03. For faces with positive labels, source memory did not differ as a function of behavior type, Δ*G*^2^(1) = 0.70, *p* = 0.40. This is clearly at odds with the assumption of a negativity advantage, and suggests that participants flexibly shifted their attention to information that was unexpected and, therefore, most informative for them.

#### Victim Sensitivity

Next, we wanted to know how guessing and source memory were affected by victim sensitivity. As reasoned in the introduction, *a priori* it seemed possible that victim-sensitive persons would show a particularly inflexible memory advantage for cheating (Gollwitzer et al., 2012). The schema violation explanation, however, would suggest that victim-sensitive persons would show stronger negative social expectations based on the negative social labels and, thus, stronger schema violation effects for trustworthy tricksters (whereas for untrustworthy scientists no difference in initial expectations due to victim sensitivity was expected).

To analyze the influence of victim sensitivity, we followed the exact same procedure as Mieth et al. (2016). Victim sensitivity was dichotomized at its sample median (i.e., 2.9). Fifty-four participants constituted the low victim sensitivity group, 50 constituted the high victim sensitivity group. These data were analyzed together using separate model trees for individuals with high and low victim sensitivity. The base model (incorporating the same restrictions as the base model reported above) fit the data well, *G*^2^(4) = 1.38, *p* = 0.85. More importantly, and in line with our expectations, participants in the high victim sensitivity group had a bias toward guessing that targets with negative labels were more likely to be cheaters than targets with positive labels, Δ*G*^2^(1) = 6.13, *p* = 0.01 (see **Table 3**).

**Table 3:** Parameter estimates of the guessing bias parameter *g* representing the conditional probability of guessing that the person was a cheater rather than a trustworthy person as a function of label and victim sensitivity (Study 1).

Label	Parameter Estimate	(*SE*)	[0.95 Confidence Interval]
**High victim sensitivity**			
Negative	0.67	(0.07)	[0.53 -0.82]
Positive	0.42	(0.06)	[0.30 -0.55]
**Low victim sensitivity**			
Negative	0.56	(0.07)	[0.42 -0.70]
Positive	0.44	(0.06)	[0.32 -0.56]

When compared against a neutral baseline of guessing with 0.50 that the target was either described as a cheater or as a trustworthy person, participants in the high victim sensitivity group had a bias toward guessing that a target with a negative label was associated with untrustworthy behavior, Δ*G*^2^(1) = 5.01, *p* = 0.03, but no bias toward guessing that a target with a positive label was associated with trustworthiness, Δ*G*^2^(1) = 1.38, *p* = 0.24. Thus, the guessing bias of victim-sensitive participants was stronger in the negative than in the positive direction. Participants in the low victim sensitivity group, in contrast, showed no such bias. Their tendency toward guessing that a target was described as a cheater (when no source memory was available) was not significantly affected by the negative or positive social label, Δ*G*^2^(1) = 1.63, *p* = 0.20.

In addition, participants in the high victim sensitivity group had enhanced source memory for violations of their label-based expectations—that is, for descriptions of trustworthiness in comparison to descriptions of untrustworthiness when the targets were associated with negative labels, Δ*G*^2^(1) = 5.54, *p* = 0.02 (see **Table 4**). As in the global analysis, there was no difference between source memory for untrustworthy and trustworthy descriptions when positive labels were used, Δ*G*^2^(1) = 0.35, *p* = 0.55. Participants in the low victim sensitivity group showed no such schema inconsistency advantage in source memory. In fact, there was no difference between untrustworthy and trustworthy descriptions, regardless of whether the labels were negative, Δ*G*^2^(1) = 0.67, *p* = 0.41, or positive, Δ*G*^2^(1) = 0.33, *p* = 0.56.

**Table 4:** Parameter estimates of the source memory parameter *d* as a function of label, behavior and victim sensitivity (Study 1).

Label	Behavior	Parameter estimate	(*SE*)	[0.95 Confidence Interval]
**High victim sensitivity**				
Negative	Untrustworthy	0.08	(0.23)	[0.00 -0.53]
Negative	Trustworthy	0.61	(0.06)	[0.50 -0.73]
Positive	Untrustworthy	0.37	(0.09)	[0.19 -0.54]
Positive	Trustworthy	0.48	(0.11)	[0.27 -0.68]
**Low victim sensitivity**				
Negative	Untrustworthy	0.39	(0.12)	[0.16 -0.62]
Negative	Trustworthy	0.54	(0.08)	[0.39 -0.68]
Positive	Untrustworthy	0.39	(0.09)	[0.22 -0.56]
Positive	Trustworthy	0.49	(0.09)	[0.30 -0.67]

In summary, Study 1 suggests that victim-sensitive compared with victim-insensitive persons have stronger negative expectations toward people associated with negative social labels, as reflected in a bias toward guessing that targets with negative labels have been associated with negative social behaviors. This finding nicely fits with prior research demonstrating that victim-sensitive individuals are more likely to use untrustworthiness cues than victim-insensitive individuals (Gollwitzer et al., 2012).

As hypothesized, the stronger initial influence of untrustworthiness cues led to an “ironic” schema inconsistency effect in the source memory of victim-sensitive individuals: behavior that was inconsistent with the negative labels was particularly well remembered as evidenced in better memory for negative targets that violated rather than confirmed negative expectations. Thus, victim sensitivity seems to be associated with a reliance on negative expectations. This reliance on negative expectations resulted in a schema-consistent guessing bias for negative labels. Moreover, the reliance on negative expectations of victim-insensitive individuals enhanced source memory for information that was inconsistent with these negative labels relative to information that was consistent therewith (indeed source memory for expectancy confirming negative targets was extremely low). Thus, whereas victim-sensitive individuals would probably prefer to remember targets well who behaved negatively (i.e., “the unstrustworthy trickster” or “the unstrustworthy scientist”), they are likely – due to strong initial negative expectations regarding targets with a negative social label – to remember those targets who surprised them by displaying positive behavior.

One limitation of the present data analytic procedure needs to be mentioned. Whereas the multinomial model is necessary to distinguish between memory and guessing, it does not allow for a direct test of interaction effects. Thus, testing “interaction effects” requires running separate models in different subgroups. The same approach has been used in previous source memory studies (Bayen and Kuhlmann, 2011; Bell et al., 2015; Mieth et al., 2016). Our present results suggest that being high in victim sensitivity is not associated with better source memory for untrustworthy behavior in general; rather, the results are more in line with the idea that victim-sensitive individuals rely on their negative schemata when guessing, and remember information that violates their negative expectations. This evidence, however, is indirect because (1) the analysis capitalized on group differences in victim sensitivity, but did not experimentally manipulate fear of exploitation, and (2) the dependent variable in Study 1 focused on the outcome of an expectancy violation, but did not measure intraindividual changes in the perception of trustworthiness after an expectancy-inconsistent vs. expectancy-consistent behavioral description has been provided.

To address these limitations, it seemed necessary to investigate the influence of victim sensitivity on the effects of violations of positive and negative expectations more directly. In Study 2, therefore, participants’ person perception rather than their source memory was examined in response to expectancy-congruent vs. expectancy-incongruent information about the targets. To foster the argument that differences in person perception are indeed causally attributable to victim sensitivity as a personality trait reflective of a latent fear of being exploited, an experimental manipulation was introduced to activate victim sensitivity.

## Study 2

### Method

#### Participants and Design

Assuming α = β = 0.05 and an effect of ϕ^2^ = 0.25 regarding the condition × victim sensitivity interaction on trustworthiness perception in a multiple regression analysis, 54 observations were needed. With a final sample size of 60, an effect of ϕ^2^ = 0.22 could be detected. The final sample consisted of 60 students (51 women, 8 men, 1 non-response) of a German university (*M*_Age_ = 22, *SD*_Age_ = 5). Participants were randomly assigned to one of two conditions, the exploitation condition (*n* = 29) or the control condition (*n* = 31; see below for details).

#### Materials

##### Fear of exploitation manipulation

Participants first read a short scenario. Their role in the scenario varied depending on experimental conditions. Participants in the exploitation condition were asked to imagine that they would have to give a presentation with two fellow students in a university course, and that they would receive a grade for their presentation which was very important to them. What follows is a summary of the scenario in the exploitation condition:

Your presentation is coming up soon and you and the two other students agree to meet in the department library after the end of the course to start with the literature search. However, the other two do not show up, and you are forced to look for the literature on your own. You eventually end up preparing the presentation all by yourself, although you tried to contact the two other students. The day before the presentation, the two others suddenly contact you and ask what their part would be in the presentation. They excuse themselves and say that they had been busy. The presentation itself works out well until the lecturer assigns grades. Your two fellow students receive better grades than you do although they barely invested anything in the presentation, and, to make matters worse, they do not object to this unjust grading. You are stupefied by the behavior of your fellow students and you feel exploited and treated unfairly.

Participants in the control condition read the same scenario, but from the perspective of an observer. Both scenarios were equal in length and varied only with regard to whether participants imagined that the event befell them (exploitation condition) or someone else (control condition). Thus, the exploitation condition should activate participants’ victim sensitivity (via imagining being exploited), whereas the control condition should not have this effect given that someone else (but not oneself) is the victim of injustice. The latter might activate participants’ observer sensitivity (Schmitt et al., 2005), but not their victim sensitivity.

##### Comprehension and manipulation check

Participants completed three items that assessed whether they had difficulties understanding the scenario (“I read the text with full concentration,” *M* = 5.70, *SD* = 0.53; “I found it easy to read the text,” *M* = 5.88, *SD* = 0.37; “I can describe the content of the text,” *M* = 5.73, *SD* = 0.48; from 1 [not at all true] to 6 [absolutely true]). As a manipulation check, participants responded to three items that assessed moral outrage and anger in response to the situation described in the scenario (“The situation described in the text makes me upset;” “The situation described in the text makes me angry;” “The situation described in the text bothers me;” from 1 [not at all true] to 6 [absolutely true]; *M* = 4.92, *SD* = 0.88, α = 0.88).

##### Trustworthiness perceptions

Next, participants viewed 24 male faces (faces, labels, and behavioral descriptions were taken from the same sources as in Study 1). Half of those were accompanied by positive social labels; the others were accompanied by negative social labels. Trustworthiness was assessed with two items (“How trustworthy is this person?” and “How likeable is this person?” from 1 [not at all] to 6 [very] that was always presented with a filler item (“How competent is this person?” from 1 [not at all] to 6 [very]).

After rating the targets’ trustworthiness for the first time (T1), participants viewed the same targets a second time (T2; in a different order). This time, the targets came with a behavioral description (trustworthy vs. untrustworthy behavior); these descriptions were also taken from the same sources as in Study 1. Nine faces with negative labels were paired with untrustworthy behavior, nine faces with positive labels were paired with trustworthy behavior, three faces with negative labels were paired with trustworthy behavior, and three faces with positive labels were paired with untrustworthy behavior.

##### Victim sensitivity

Finally, participants’ victim sensitivity was assessed with the same 10-item scale as in Study 1 (Schmitt et al., 2010; *M* = 4.23, *SD* = 0.69, α = 0.80). Participants’ victim sensitivity did not differ as a function of the experimental manipulation, *t*(58) = 0.75, *p* = 0.45.

### Results and Discussion

#### Manipulation Check

To test whether the experimental manipulation was successful, a hierarchical regression analysis was conducted with condition (0 = control, 1 = exploitation), victim sensitivity (mean-centered), and the condition × victim sensitivity interaction (entered in a second step) as predictor variables, and moral outrage about the scenario as dependent variable. Neither victim sensitivity, *B* = 0.28, *t*(57) = 1.72, *p* = 0.09, nor condition, *B* = 0.19, *t*(57) = 0.91, *p* = 0.40, had main effects on moral outrage, but the interaction effect was significant, *B* = 0.69, *t*(56) = 2.20, *p* = 0.03, Δ*R*^2^ = 0.074. We probed this interaction using Hayes’s (2013) PROCESS macro. For people high in victim sensitivity (1 *SD* above the sample mean), moral outrage scores were significantly higher in the exploitation than in the control condition, *B* = 0.67, *t*(56) = 2.18, *p* = 0.03, whereas for people low in victim sensitivity (1 *SD* below the sample mean), the difference between the two experimental conditions was not significant, *B* = -0.29, *t*(56) = -0.94, *p* = 0.35. Stated differently, victim sensitivity predicted moral outrage in the exploitation condition, *B* = 0.59, *t*(56) = 2.79, *p* = 0.007, but not in the control condition, *B* = -0.10, *t*(56) = -0.44, *p* = 0.66. Thus, the experimental manipulation was successful in activating victim sensitivity.

#### Trustworthiness Perceptions at T1

Participants’ trustworthiness perceptions were aggregated (1) across the nine expectancy-confirming positive targets (positive label plus trustworthy behavioral description), (2) across the 9 expectancy-confirming negative targets (negative label plus untrustworthy behavioral description), (3) across the three expectancy-violating positive targets (positive label plus untrustworthy behavior), and (4) across the three expectancy-violating negative targets (negative label plus trustworthy behavior). Mean trustworthiness perceptions are displayed in **Table 5**.

**Table 5:** Descriptive findings for the trustworthiness perceptions at T1 and change in perceived trustworthiness at T2 (Study 2).

Measure		*N* Items	Mean	*SE*
Label	Behavior			
**Trustworthiness perceptions at T1**				
Negative		18	2.62	0.71
Negative		6	2.92	0.86
Positive		18	4.25	0.57
Positive		6	4.16	0.71
**Difference score (T2-T1)**				
Negative	Untrustworthy	18	-1.37	0.61
Negative	Trustworthy	6	1.59	0.70
Positive	Untrustworthy	6	-2.82	0.78
Positive	Trustworthy	18	0.95	0.41

**FIGURE 2:**
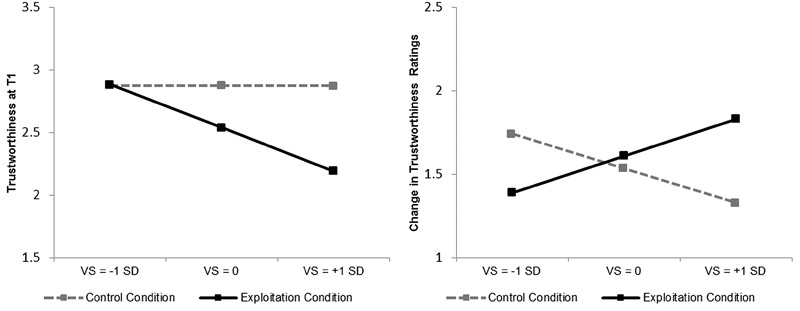
**Effect of victim sensitivity and exploitation condition on initial trustworthiness perception **(left)** and change in trustworthiness **(right)** for targets with a negative social label and a positive behavioral description.** Higher values on change in trustworthiness reflect stronger change toward more trustworthiness. Note that victim sensitivity was included as a continuous predictor in all analyses. Plotting the dependent variables at three levels of victim sensitivity only serves to visualize the findings. VS = victim sensitivity.

The effects of experimental condition and victim sensitivity on trustworthiness perceptions at T1 (i.e., without behavioral descriptions) were tested via hierarchical multiple regression analysis with condition (0 = control, 1 = exploitation), victim sensitivity (mean-centered), and their interaction term (entered in a second step) as predictors. These analyses were conducted for targets with positive and targets with negative labels, respectively. For targets with positive labels, neither the experimental manipulation nor victim sensitivity (or their interaction) influenced trustworthiness perceptions, all *p*s > 0.42. For targets with negative social labels, however, trustworthiness perceptions were significantly affected by victim sensitivity, *B* = -0.28, *t*(57) = -2.16, *p* = 0.03, Δ*R*^2^ = 0.071, condition, *B* = -0.33, *t*(57) = -1.90, *p* = 0.06, Δ*R*^2^ = 0.055, and their interaction, *B* = -0.50, *t*(56) = 1.99, *p* = 0.05, Δ*R*^2^ = 0.057 (see **Figure 2**, left panel). Probing this interaction further showed that people high in victim sensitivity (1 *SD* above the sample mean) gave significantly lower trustworthiness perceptions in the exploitation than in the control condition, *B* = -0.68, *t*(56) = -2.78, *p* = 0.007, whereas for people low in victim sensitivity (1 *SD* below the sample mean), this effect was not significant, *B* = 0.01, *t*(56) = 0.03, *p* = 0.97. Stated differently, victim sensitivity predicted lower trustworthiness perceptions in the exploitation condition, *B* = -0.50, *t*(56) = -2.98, *p* = 0.004, but not in the control condition, *B* = -0.00, *t*(56) = -0.01, *p* = 0.99.

#### Changes in Trustworthiness Perceptions

To quantify participants’ responsiveness to behavioral descriptions, a difference score was computed by subtracting trustworthiness perceptions at T1 (without behavioral descriptions) from their trustworthiness perceptions at T2 (with behavioral descriptions). Thus, positive values on the difference score reflect an increase in perceived trustworthiness at T2 relative to T1; negative values reflect a decrease. Multiple regression analyses (see above) were conducted to analyze the effect of our predictor variables on expectancy-violating targets.

Regarding expectancy-violating targets with a *positive* social label (e.g., untrustworthy scientists), changes in trustworthiness perceptions were neither predicted by condition, nor by victim sensitivity, nor their interaction, all *p*s > 0.44. However, regarding expectancy-violating targets with a *negative* social label (e.g., trustworthy tricksters), changes in perceived trustworthiness were significantly predicted by the condition × victim sensitivity interaction, *B* = 0.62, *t*(56) = 2.37, *p* = 0.02, Δ*R*^2^ = 0.091 (see **Figure 2**, right panel). Probing this interaction further revealed that people high in victim sensitivity (1 *SD* above the sample mean) showed significantly greater change in perceived trustworthiness in the exploitation compared to the control condition, *B* = 0.50, *t*(56) = 1.98, *p* = 0.05, whereas for people low in victim sensitivity (1 *SD* below the sample mean), this effect was not significant, *B* = -0.35, *t*(56) = -1.39, *p* = 0.17.

In a final step, we tested whether victim-sensitive compared with victim-insensitive individuals indeed update their expectations particularly when a target with a negative social labels turns out to be trustworthy. To do so, a mixed model was performed on participants’ absolute change scores regarding their trustworthiness perception of negative targets who violated versus confirmed negative expectations. No restriction was imposed on the covariance matrix and parameters were estimated using full maximum likelihood. This analysis yielded a significant three-way interaction between type of target (confirming vs. violating) × condition × victim sensitivity (*p* = 0.008): Victim-sensitive individuals in the exploitation condition updated their perceptions of negative targets who violated their expectations (see the just reported results of the multiple regression analyses for the direction of this updating), but not of negative targets who confirmed them. Indeed, for negative targets who confirmed negative expectations victim-sensitivity was related to reduced updating of perceptions in the exploitation condition, *B* = -0.38, *t*(52) = -2.66, *p* = 0.01, whereas in the control condition victim sensitivity was unrelated to changes in trustworthiness toward such targets, *B* = -0.02, *t*(52) = -0.10, *p* = 0.92.

Thus, although participants high in victim sensitivity tend to distrust targets with negative social labels initially, they are more likely to selectively update their trustworthiness perception after receiving expectancy-violating information relative to participants low in victim sensitivity. This greater change in perceived trustworthiness seems to reflect that, when fear of exploitation is high, people are particularly responsive to the violation of negative expectations. Moreover, the fact that victim sensitivity predicted greater change in trustworthiness in the condition that activated participants’ victim sensitivity is in line with the argument that the observed sensitivity to a violation of negative expectations is indeed causally attributable to differences in participants’ fear of exploitation.

## General Discussion

When people are victim-sensitive, they are more receptive toward cues associated with untrustworthiness, such as the interaction partner’s facial expression or his or her background. So, when fear of exploitation is high, negative social labels such as “X is a trickster” have a stronger influence on one’s trustworthiness perception of X than a positive social labels such as “X is a scientist.” This has been suggested by recent research on victim sensitivity and suspicious cognition (see Gollwitzer and Rothmund, 2009; Gollwitzer et al., 2013, for theoretical reviews). The present study corroborates and extends these findings by asking: what happens if an initial expectation regarding a particular interaction partner is violated, that is, if a “trickster” turns out to be trustworthy rather than untrustworthy? Here, a “cheater detection” account (Cosmides and Tooby, 1989) would predict that people are more likely to attend to (and remember) the latter information. But recent research suggests that memory advantages for cheaters are not as robust as evolutionary psychology thought they would be. Source memory effects can be better conceptualized as expectancy-violation effects than as cheater detection effects (Bell and Buchner, 2012). Thus, it was hypothesized that participants with a fear of exploitation would not show enhanced memory accuracy for the untrustworthy behavior of the trickster because untrustworthiness is already part of their negative stereotype of a trickster. In contrast, they should be more influenced by information that contradicts their initial (negative) expectation. This asymmetric effect should manifest in (a) better source memory and (b) increased changes in trustworthiness perceptions for a negatively labeled target that shows trustworthy behavior (i.e., the “trustworthy trickster”) compared to a negatively labeled target that turns out to be a cheater (i.e., the “untrustworthy trickster”). The results of the two studies described in this paper confirm this reasoning and, thus, contribute to and qualify research on trustworthiness, suspiciousness, and source memory in social interactions.

### Victim Sensitivity and Asymmetric Attendance to Untrustworthiness Cues

In previous studies (Suzuki and Suga, 2010; Bell et al., 2012), it has been demonstrated that people rely on cues of untrustworthiness in a person’s facial appearance if they do no longer remember a person’s previous behavior. Consistent with these findings, the social labels affected participants’ guessing behavior in Study 1. If source memory was no longer available, participants guessed that targets associated with negative social labels had been associated with untrustworthy behavior more often than guessing that targets associated with positive social labels had been associated with trustworthy behavior. This effect was only found among participants who were classified as high in victim sensitivity and was not evident among participants low in victim sensitivity. This finding confirms and expands previous research on victim sensitivity, which showed that victim-sensitive individuals are more likely than victim-insensitive individuals to attend to social cues associated with untrustworthiness rather than trustworthiness (Gollwitzer et al., 2012). The crucial difference between this previous research and the present experiments is that previous research on asymmetrical attendance to untrustworthiness cues solely relied on self-reports (about another person’s trustworthiness), whereas the first study in the present article obtained evidence for this effect in a much more unobtrusive measure: participants’ guessing in the absence of source memory. This finding lends support to the “asymmetry hypothesis” formulated by Gollwitzer et al. (2013) in their “Sensitivity to Mean Intentions” (SeMI) model.

### Victim Sensitivity and Asymmetrical Effects on Expectancy-Inconsistent Information

The finding that people high in victim sensitivity have a guessing bias toward untrustworthiness after being confronted with untrustworthiness-related cues is interesting in itself. However, it takes us even one step further by answering the question how people who fear to be exploited react to information that violates their initial expectation about the trustworthiness of their interaction partners.

Notably, the SeMI model does not make straightforward predictions about how victim-sensitive individuals should respond to persons who violate or confirm their initial expectations. Considering that victim-sensitive individuals experience particularly strong negative emotions when they are exploited, it seemed possible that victim sensitivity may lead to an inflexible memory advantage for cheaters. In other words, it seemed possible that victim-sensitive compared with victim-insensitive individuals recall that somebody turned out to be a cheater more easily than the information that somebody turned out to be a nice person.

In general, both types of information—the information that someone is a cheater as well as the information that someone is trustworthy—helps to decrease social uncertainty which is experienced as aversive by victim-sensitive individuals. When such information is available, remembering expectancy-inconsistent information may be particularly useful for social exchange. For instance, when people are in a situation in which cooperation is low and cheating is common, they may be extremely reluctant to cooperate with people whose previous behavior is unknown. In this situation, it is not helpful for an individual to remember particular instances of cheating because, with or without this information, this individual will refuse to cooperate (Barclay, 2008; Bell et al., 2010). Instead, it seems more functional to focus on those few cases in which the behavior is inconsistent with one’s expectations about a person. Given that the effect of schema inconsistency on memory has been shown to be a fairly general phenomenon (Bell and Buchner, 2012), it seems possible that victim-sensitive individuals—due to their increased negative expectations—may show increased processing of information that specifically violates their *negative* views of other persons.

In line with this latter prediction, participants in Study 1 demonstrated better source memory for behaviors that were inconsistent with negative expectations than for behaviors that were consistent with these expectations. This finding cannot be explained by a cheater detection account, but it can be explained by an expectancy inconsistency account. Moreover, Study 1 demonstrated that this expectancy inconsistency effect was particularly evident for people high in victim sensitivity. These people, however, showed no memory advantage for behaviors that violated positive stereotypes, which, at first glance, seems to be at odds with the SeMI model: according to this model, realizing that somebody is cheater although one expected this person to be trustworthy should be particularly aversive to victim-sensitive relative to victim-insensitive individuals. Interestingly, prior research (Bell and Buchner, 2010) found that observer-sensitive individuals (i.e., people with a true concern for the just treatment of others) do have better source memory for cheaters. In light of the present results and using an expectancy inconsistency account of memory, this prior finding might be better understood: whereas people high compared with people low in victim sensitivity have biased expectations toward targets associated with untrustworthiness, people high compared with people low in observer sensitivity might harbor more positive expectations toward targets associated with trustworthiness. Hence, learning that the scientist is untrustworthy constitutes a greater expectancy violation for the person high in observer sensitivity, whereas learning that the trickster is trustworthy constitutes a greater expectancy violation for the person high in victim sensitivity (resulting in the findings presented here and the ones observed by Bell and Buchner, 2010).

Results from Study 2 extended the findings observed on participants’ source memory to participants’ trustworthiness perceptions and provided experimental evidence in that regard. In this study, victim-sensitive individuals whose fear of exploitation was experimentally activated were more likely to update their trustworthiness perceptions if a negatively labeled target turned out to display trustworthy behavior. The opposite effect, that is, updating trustworthiness ratings for a positively labeled target who turned out to be a cheater, was not influenced by being victim-sensitive or victim-insensitive in Study 2. Thus, our results can be summarized as follows: victim-sensitive individuals show asymmetric expectancy violation effects which is evidenced in an asymmetric memory advantage for schema-inconsistent information as well as an asymmetric change in person perception.

### Limitations

In the present studies, participants observed targets. Thus, it is not clear whether similar effects on memory and impression updating would be obtained if participants were the actual recipients of trustworthy versus untrustworthy behavior. However, research on cooperation in public goods games demonstrated that victim sensitivity is a powerful predictor of withholding contributions when cues of exploitation are present (Gollwitzer and Rothmund, 2011). Therefore, there is good reason to assume that victim sensitivity in terms of a fear of exploitation does drive expectations and expectation violations also in more interactive situations.

Another potential limitation pertains to the operationalization. Participants only judged male targets. Hence, it is unclear whether similar effects would be obtained for female targets. Moreover, the majority of participants were female. However, gender has only very small effects on victim sensitivity (η^2^ = 0.002 in the validation study by Schmitt et al., 2010).

Finally, the present research demonstrates that victim-sensitive individuals react more strongly to certain types of expectancy violations. However, it is unclear which processes involved in expectancy violations drive the observed effects. Thus, whereas a purely cognitive process is possible in which victim sensitivity exacerbates contrast effects by increasing the difference in valence of the elements involved in the comparison (Biernat, 2005), it is also conceivable that victim sensitivity is related to greater feelings of surprise following expectancy evaluations. Importantly, stronger surprise alone might suffice to exacerbate contrast effects as it stimulates stronger sense-making and cognitive mastering (see Noordewier et al., 2016 for a temporal dynamics account of surprise).

## Conclusion

In conclusion, the present research supports the hypothesis that victim sensitivity and therewith a fear of exploitation need not result in an increase in response to cheating but may ironically increase the processing of information that is inconsistent with negative stereotypes and expectations. This finding cannot be accounted for by a cheater detection explanation but nicely fits an expectancy violation account—in which victim sensitivity systematically affects participants’ initial schematic expectations for dubious targets leading to stronger effects in participants’ source memory and trustworthiness perceptions if these expectations are not met. This might in fact be interpreted as good news, as it suggests that even persons with a high fear of exploitation are able to overcome their habitual negative expectations toward their social environment when they are confronted with more valid information about another person’s trustworthiness.

## Ethics Statement

The reported studies were exempt from requiring an approval of an ethics committee. Studies were voluntary, anonymous, non-invasive, and did not involve deception. Approval of an ethics committee for such studies is not an requirement in Germany where the studies were conducted. Participants received general information about the study before taking part, were informed that participation is voluntary, and that participation can be withdrawn.

## Author Contributions

PS drafted the manuscript. MG, LM, AB, and RB revised the manuscript. Study 1 was designed and analyzed by LM, AB, and RB. Study 2 was designed and analyzed by PS and MG.

## Conflict of Interest Statement

The authors declare that the research was conducted in the absence of any commercial or financial relationships that could be construed as a potential conflict of interest.

## Footnotes

1 The hypothesis that *D*_C_ = *D*_T_ is supported by the available literature (e.g., Barclay and Lalumière, 2006; Buchner et al., 2009), as well as by the present results. Furthermore, the statistical comparisons do not change when using a base model that does not make this assumption and simply assumes that *D*_New_ = (*D*_C_ + *D*_T_)/2 (as in Bell et al., 2015).
